# New dawn for keratoconus treatment: potential strategies for corneal stromal regeneration

**DOI:** 10.1186/s13287-023-03548-5

**Published:** 2023-11-06

**Authors:** Shengqian Dou, Xiaoxue Liu, Weiyun Shi, Hua Gao

**Affiliations:** 1 State Key Laboratory Cultivation Base, Shandong Provincial Key Laboratory of Ophthalmology, Eye Institute of Shandong First Medical University, Qingdao, China; 2 Qingdao Eye Hospital of Shandong First Medical University, Qingdao, China; 3 Eye Hospital of Shandong First Medical University, Jinan, China; 4 School of Ophthalmology, Shandong First Medical University, Jinan, China

**Keywords:** Keratoconus treatment, Corneal stromal regeneration, Cell therapy, Biosynthetic alternatives, Minimally invasive intrastromal implantation, Bioengineered tissues

## Abstract

Keratoconus is a progressive, ectatic and blinding disorder of the cornea, characterized by thinning of corneal stroma. As a highly prevalent among adolescents, keratoconus has been a leading indication for corneal transplantation worldwide. However, the severe shortage of donor corneas is a global issue, and the traditional corneal transplantation surgeries may superinduce multiple complications, necessitating efforts to develop more effective strategies for keratoconus treatment. In this review, we summarized several strategies to promote corneal stromal regeneration or improve corneal stromal thickness, including cell-based therapies, biosynthetic alternatives for inducing corneal regeneration, minimally invasive intrastromal implantation and bioengineered tissues for implantation. These strategies provided more accessible but safer alternatives from various perspectives for keratoconus treatment, paving the way for arresting the keratoconus progression in its earlier stage. For the treatments of corneal ectatic diseases beyond keratoconus, these approaches will provide important references and widen the therapy options in a donor tissue-independent manner.

## Background

Keratoconus is a progressive corneal ectatic disorder characterized by thinning of corneal stroma and asymmetrical conical protrusion of the cornea, which can lead to visual impairment or even blindness [1–3]. Keratoconus is one of the leading indications for corneal transplantation surgery worldwide [4, 5], with an incidence of 1/2000 in the general population and even higher among young adults [2, 6]. Keratoconus is the result of complex genetic and environmental interactions [7–9]. The most severe stage of keratoconus manifests with excessive ectasia, scarring and thinning stroma, which significantly impairs the vision, and the only option left for patients is corneal transplantation [1]. However, the severe shortage of the donor corneas available for transplant represents a global burden of blindness, with one cornea available for every 70 recipients in waiting [10]. Besides, traditional corneal transplantation surgeries can cause various complications, such as the severed corneal nerve plexus, dry eye, glaucoma and tissue rejection. Due to the immune rejection and chronic corneal allograft dysfunction, the poor long-term graft survival rate after keratoplasty usually brings a huge burden on patients. For these reasons, intense research effort has focused on corneal stromal regeneration to increase the corneal thickness of patients with keratoconus, and multiple therapy paradigms have been explored as alternative treatment modalities to preserve and improve the vision [11–14]. In this review, the strategies for corneal stromal regeneration are summarized, highlighting potential approaches for keratoconus treatment.

## Strategies for corneal stromal regeneration

### Cell therapy for keratoconus treatment

Currently, corneal collagen cross-linking and corneal transplant remain the most preferred or even the only option for keratoconus treatment. However, neither of these approaches can fundamentally solve the underlying issue of the disease. Approximately 80–85% of the corneal thickness is composed of the corneal stroma, in which collagen fibrils and extracellular matrix are tightly arranged [15, 16]. Keratocyte loss and excessive degradation of collagen fiber by matrix metalloproteinases are the culprit of keratoconus pathogenesis [17, 18]. Hence, replacing or reviving the corneal stromal cells might be an ideal and direct approach; therefore, cell-based therapies for corneal stromal regeneration during keratoconus treatment have emerged and gained great concern.

**Fig. 1:**
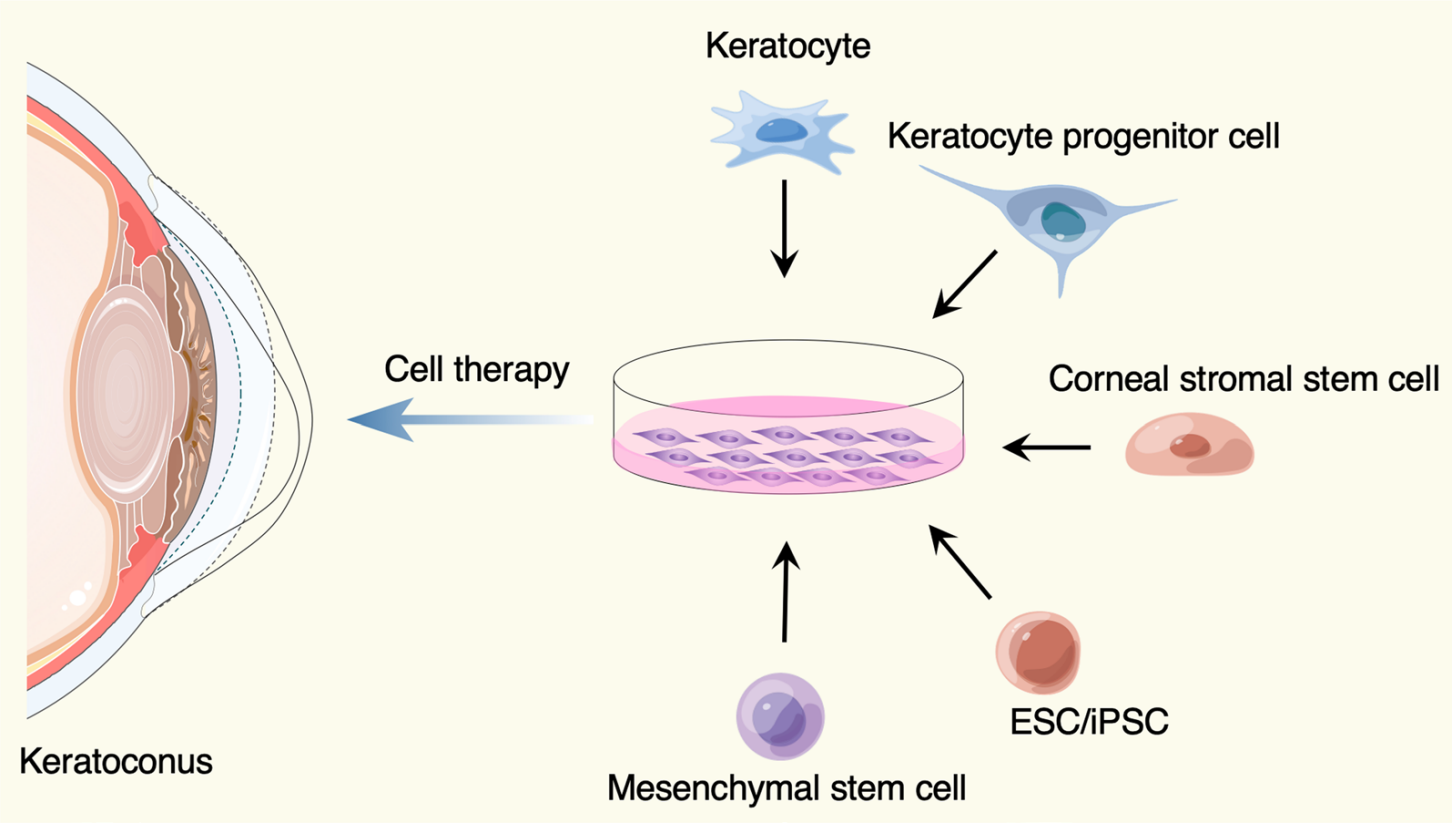
Cell sources used for keratoconus treatment. The figure was prepared by our group, and some of the elements in the diagram were provided by Figdraw (http://www.figdraw.com)

To date, various ideas and choices for cell therapy of keratoconus were developed (Fig. 1, Table 1). Keratocytes in the cornea are derived from neural crest cells. The number of keratocytes are limited in vivo, but they can be cultured in vitro and supplied as reliable cell source for intrastromal injection [19, 20]. Besides, keratocyte progenitor cells, the committed stem cell populations that maintain capacity to self-renewal and differentiation, are thought to be a potential option for keratoconus treatment. The transplantation of healthy keratocyte progenitor cells into keratoconus corneas would provide a novel treatment modality that may slow the progression of keratoconus [21]. Moreover, the corneal stromal stem cells, a rare cell population resident in the peripheral cornea and limbus, can be isolated by specific surface markers from limbal stromal tissues [22–25]. Du et al. injected the human corneal stromal stem cells into mice corneas and did not observe elicit immune rejection over an extended period of time, suggesting an opportunity to develop cell-based therapies for corneal stromal diseases [26].

**Table 1:** A summary of treatment approaches for corneal stromal regeneration

Categories	Cell/tissue sources	Therapeutic method	Stage of research	Advantages	Limitations
Cell-based therapies	Keratocytes from corneal stroma [20]	Intrastromal injection	Animal studies	A nonsurgical approach to revive the keratocyte population	Limited numbers; further work is needed
	Keratocyte progenitor cells from limbal rings [21]	Introduction into stroma via femtosecond laser channels	Hypothesis	The corneal rims which are usually discarded after keratoplasty, are potentially a plentiful source	Its safety and feasibility need to be explored
	Corneal stromal stem cells from limbal rings [22–25]	Engrafted in a fibrin gel on ocular surface	Animal studies	A nonsurgical approach to promote stromal regeneration and prevent scarring	Limited numbers; further work is needed for keratoconus treatment
	Adipose tissue [30, 31, 36]	Prepared ADSCs were placed into the mid-stromal pocket	Phase I clinical study	Autologous cells; adipose tissue is abundant and easily accessible	More clinical testing should be down
	Hematopoietic stem cells [32]	Intrastromal injection	Animal studies	Autologous cells	Further studies are needed
	Dental pulp [35]	Intrastromal injection	Animal studies	Autologous cells; adequate sources	Further studies are needed
	Umbilical cord blood [33, 34]	Intrastromal injection	Animal studies	Adequate sources	Further work is needed for keratoconus treatment
	iPSCs [38]	Intrastromal injection	Cell lines	Adequate sources	Further studies are needed
Biosynthetic alternatives	RHCIII [39]	Implantation	Phase I clinical study	A safe and effective alternative to be implanted to help address the donor cornea shortage	More patients, modified surgical techniques and continued biomaterials development are needed
	Short collagen-like peptides conjugated to polyethylene glycol [41]	Implantation	Preclinical mini-pig model	A sufficiently robust for surgical handling implant which provide stable corneal tissue regeneration	More clinical testing should be down
	LiQD cornea [42]	A cell-free, liquid hydrogel matrix applied on ocular wounding surface	Animal studies	Convenient, less costly and reduces the risk of immune rejection risk	More clinical testing should be down

However, several cell types mentioned above are still dependent on the corneal tissues, and the shortage of donor corneal tissues and the limited numbers of the particular cell populations is a significant challenge. The corneal stromal cells were found to have properties similar to other mesenchymal stem cells from various tissues [24, 27, 28], including adipose tissue [29–31], hematopoietic stem cells [32], dental pulp [33, 34] and umbilical cord blood [35], which have been demonstrated to be used for keratoconus cell therapy [11]. For example, implantation of autologous adipose tissue-derived stem cells (ADSCs) into corneal stroma has been successfully tested for the treatment of keratoconus [30, 31, 36, 37]. In addition, embryonic stem cells (ESCs) and induced pluripotent stem cells (iPSCs) also provide sufficient cell sources that could be differentiated to keratocytes required for keratoconus therapy [38].

### Biosynthetic alternatives for inducing corneal regeneration

Replacement of the damaged tissue with corneal transplants is widely accepted treatment for corneal blindness. Over ten years ago, Per Fagerholm et al. developed a kind of recombinant human collagen type III (RHCIII), which has undergone synthesized in yeast, chemically cross-linked, and molded into a biosynthetic cornea mimic [39]. They conducted a phase 1 clinical study in which the biosynthetic cornea mimics were implanted to replace the distorted corneas of 10 patients with keratoconus or central scar. Strikingly, corneal re-epithelialization occurred in all patients, and nerve regeneration and touch sensitivity were also restored, demonstrating the property of the biosynthetic mimics in facilitating endogenous tissue regeneration. After then, further optimization of the biosynthetic corneal implants was done [40, 41]. More significantly, Christopher D. McTiernan et al. developed a regeneration-stimulating liquid corneal replacement in a syringe that gels in situ, LiQD cornea, that comprises short collagen-like peptides, polyethylene glycol and fibrinogen [42]. The self-assembling synthetic collagen analog, as a low-cost and immune-compatible alternative, offering a safe and effective option to help address the current donor cornea shortage. Detailed information of these approaches is listed in Table 1.

## Mechanical methods to improve corneal stomal thickness

### Minimally invasive intrastromal implantation

Beside the strategy of corneal stromal cell replacement, restoring the physical properties of the corneal stroma cannot be ignored during keratoconus treatment [14]. Substantial biomechanical imbalance and weakening of the cornea can distinctly deteriorate the ocular surface homeostasis [43, 44]. As we know, eye-rubbing is one of the major risk factors for keratoconus progressive, which can induce distinct alterations in corneal biomechanics [45–47]. And mechanical stretch was a trigger for keratoconus development and biomechanics-enzymes axis played a pathogenic role in keratoconus, as identified in our study [48]. Therefore, strengthening the biomechanical properties of the cornea should be considered during keratoconus treatment.

**Fig. 2:**
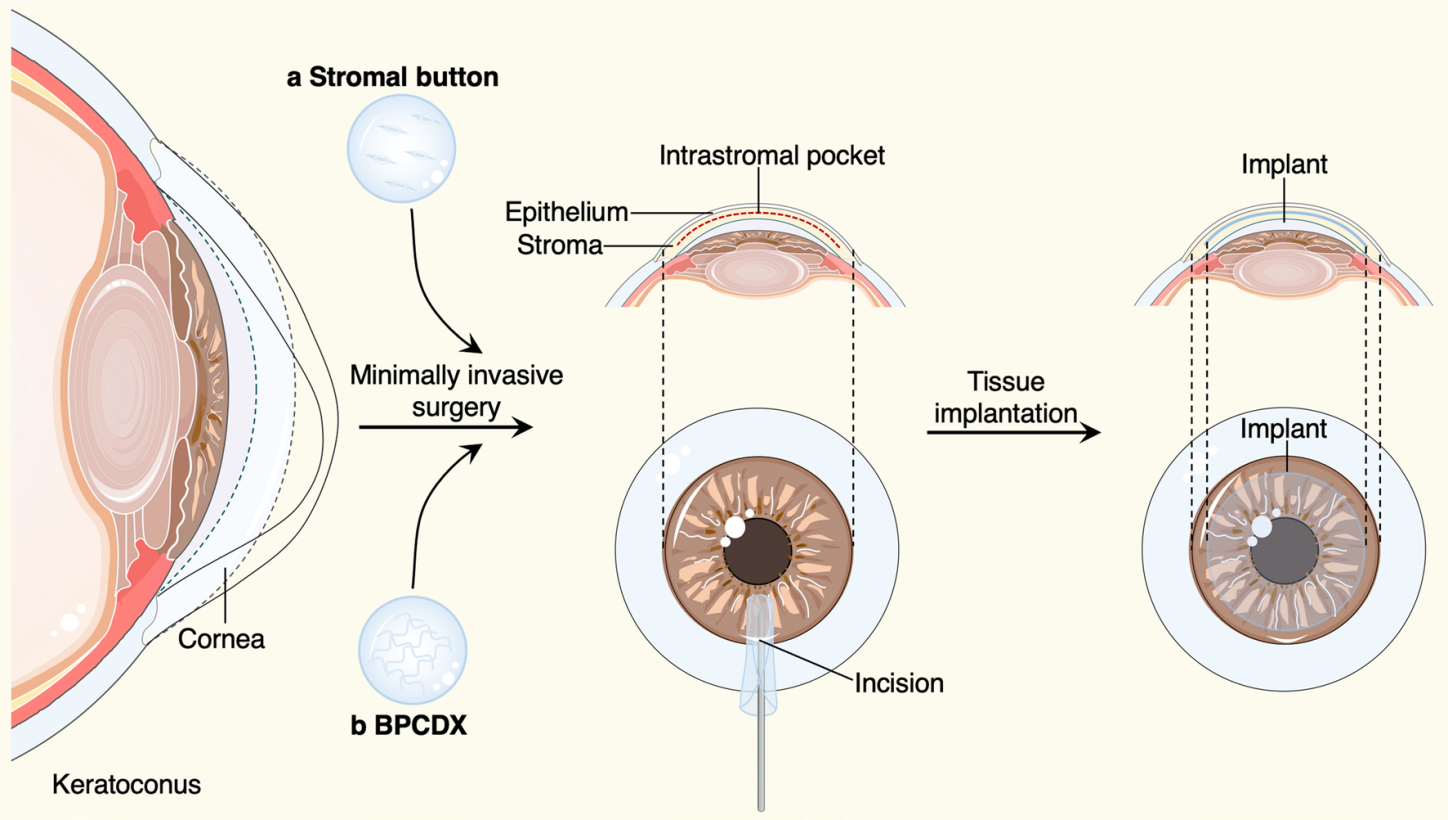
Minimally invasive surgical methods and bioengineered grafts for keratoconus treatment. An intrastromal pocket with a small incision were created by femtosecond laser, the human stromal button (**a**) or bioengineered BPCDX graft (**b**) were gently inserted into the intrastromal pocket to increase the corneal thickness. The figure was prepared by our group

In recent years, corneal collagen cross-linking therapy, as a primary operative correction for progressive keratoconus, are used routinely to increase the biomechanical stability of the cornea. However, for keratoconus that has progressed to the most severe stage, corneal transplantation is the only option [1] (Table 2). Penetrating keratoplasty (PK) is a transplant procedure with full-thickness resection of the cornea, followed by grafting it with a full-thickness donor cornea, which was the treatment of choice for keratoconus until the late twentieth century [49–51]. When indicated, refinements in surgical approaches, like the deep anterior lamellar keratoplasty (DALK) [49–51] and anterior lamellar keratoplasty (ALK) [52], that were surgical procedures for removing part of the cornea. For instance, DALK involves replacement of the pathological corneal stroma down to the Descemet’s membrane but with the functional corneal endothelium retained, offers an effective alternative procedure that may lessen the risks including graft rejection and irregular astigmatism in PK. Despite DALK's success in restoring keratoconus patients' vision, there is still room for improvement regarding the operational complexity, restoring the physical properties of corneal stroma, preservation of the anterior corneal structure and nerve plexus, and suture-related complications. Therefore, suture-free implementation with smaller access cuts may be a preferred surgical option to arrest the progress of keratoconus, such as epikeratophakia (EP) [53, 54], Bowman layer (BL) transplantation [55, 56] and allogenic lenticule implantation [57–60]. Besides, our group have introduced a new effective procedure for the treatment of advanced keratoconus, named “femtosecond laser-assisted minimally invasive lamellar keratoplasty” (FL-MILK), in which partial thickness corneal stroma (stromal button) was implanted to the allogeneic corneal stroma through a small incision created by femtosecond laser (intrastromal pocket) [61] (as illustrated schematically in Fig. 2). Our study also indicated that FL-MILK can stabilize progressive KC in mild-to-moderate cases and advanced cases at 24-month follow-up with sustainable flattening effect of the anterior cornea curvature [62]. Indeed, while improving the stromal thickness, this minimally invasive surgical methods can maximally maintain the structural integrity and physical properties of the cornea, providing a feasible option for keratoconus treatment that should be put on the agenda.

**Table 2:** A summary of mechanical approaches to improve corneal stomal thickness

Categories		Therapeutic opportunity	Stage of research	Advantages	Limitations
Surgical methods	PK [49–51]	Advanced keratoconus	In clinical applications	Better visual outcomes	Higher rates of graft rejection, suture-related complications, high residual astigmatism; poor long-term graft survival rate
	DALK [49–51]	Stage II–III	In clinical applications	Reduced rejection and astigmatism	Complexity of operation; risks of suture-related complications
	ALK [52]	Advanced keratoconus	In clinical applications	Lower risk of graft rejection; less endothelial cell loss; better graft survival; less intra-/postoperative complications	Suture-induced complications
	FL-MILK [61]	Stage II-III	In clinical applications	Plentiful source; minimally invasive, more precise and quick recovery	It needs a longer follow-up period to answer the concerns about the maintenance of stabilizing effects
	EP [53, 54]	Stage I-II	In clinical applications	A safe and minimally invasive extraocular procedure; no interference with later surgical treatment	Ectatic changes; abnormal epithelial cell ingrowth
	BL transplantation [55, 56]	Advanced keratoconus	In clinical applications	It can postpone or prevent a more invasive corneal surgery, while minimizing the complications and allowing less stringent surveillance and intensive medical therapy	Patients should be counseled to avoid eye-rubbing; allergies may need closer monitoring and treatment; limited increase in corneal thickness; the long-term remissive effect is uncertain
	Allogenic lenticule Implantation [57–60]	Stage II-III	In clinical applications	It eases the harvesting of the graft; minimally invasive	More cases and longer follow-up are needed for keratoconus treatment
Bioengineered corneal tissues	DPC [64]	–	In clinical applications	Plentiful source; low risk of graft rejection	Conventional suture methods are not applicable; further explorations are needed for keratoconus treatment
	BPCDX [65]	Advanced keratoconus	In clinical trials	Plentiful source; safer, simpler and minimally invasive method	Ethical issues remain to be defined; more clinical testing should be down

### Bioengineered corneal tissues for implantation

For the keratoplasty to treat keratoconus, several materials can be used as biomedical implants. The natural cornea has particular advantage in mechanical properties and structures, while the severe shortage of donor corneas presents a global concern. Hence, intense research efforts have focused on effective alternatives to conventional corneal grafts. May Grifith et al*.* successfully multilayered corneal equivalents constructed from immortalized cell lines [63]. Per Fagerholm et al*.* conducted a phase 1 clinical study in which biosynthetic mimics of corneal extracellular matrix were implanted to induce corneal regeneration [39]. Our group have developed a protective decellularization strategy for the preparation of decellularized porcine cornea (DPC), which achieved equivalent levels in numerous properties compared with that of human cornea grafts [64]. All these studies offered prospects for visual rehabilitation of corneal blindness. Even more to the point, Mehrdad Rafat and colleagues have described a cell-free engineered corneal tissue, which was derived from purified type I porcine collagen with dual chemical and photochemical cross-linking applied, termed the bioengineered porcine construct, double cross-linked (BPCDX) [65] (Fig. 2). The authors extracted and purified collagen from a by-product of the food industry, the porcine skin, providing an abundant yet sustainable and cost-effective supply of raw materials for implants. At the same time, likewise, the authors insert the implant within the corneal stroma through a minimally invasive procedure. Notably, after 2 years of follow-up, no adverse event was reported, all participants' vision improved to the same degree as with a standard donor tissue transplant. The strategy proposed by this work in which accessible bioengineered corneal tissues and minimally invasive surgical methods were elaborately combined, would be an attractive option for treatment of advanced keratoconus, especially in resource-limited settings. Details of the approaches mentioned in this part are listed in Table 2.

## Discussion

In this review, we summarized several approaches to promote corneal stromal regeneration or improve corneal stromal thickness, including cell-based therapies, biosynthetic alternatives for inducing corneal regeneration, minimally invasive intrastromal implantation and bioengineered tissues for implantation. Among these, a series of mechanical methods to improve corneal stomal thickness have been applied in clinical treatment of keratoconus. For instance, historically, PK has been the gold standard approach for the surgical treatment of advanced keratoconus with its good visual outcomes [50, 51]. However, DALK is increasingly becoming the preferred primary surgical option in contemporary practice owing to its reduced rejection and astigmatism in PK complications. But the complexity of operation and risks of suture-related complications in DALK complications cannot be ignored, which prompted the occurrence of minimally invasive surgical methods [49–51]. For example, FL-MILK can maximally maintain the structural integrity and physical properties of the cornea while improving the stromal thickness, and its more precise and quick recovery might make it an effective alternative for the treatment of advanced keratoconus [61]. Besides, combined more accessible bioengineered corneal tissues and minimally invasive methods would be an attractive option for keratoconus treatment. Indeed, longer follow-up period and more cases are needed for several new improving approaches.

In addition, the severe shortage of donor tissue impeded the treatment of keratoconus through corneal transplant surgery, especially in resource-limited settings. Therefore, explorations in developing strategies to promote corneal stromal regeneration has never stopped. The ideal cell-based therapy is expected to replace or revive the diseased keratocyte cells by inducing regeneration or by exogenous transplantation of keratocyte-committed cells. Here we listed the cell sources, stage of research, advantages and limitations for various cell-based therapeutic methods. Among these, implantation of autologous ADSCs into corneal stroma has been successfully tested for the treatment of keratoconus in clinical trials [30, 31, 36, 37], with its abundant and easily accessible cell source. Besides, biosynthetic alternatives for inducing corneal regeneration, including RHCIII [39] and LiQD cornea [42], providing low-cost and immune-compatible alternatives to help address the donor cornea shortage.

## Conclusions

Collectively, this review highlighted the advances in therapeutic strategies that can promote corneal stromal regeneration or improve corneal stromal thickness for keratoconus treatment, providing important reference and foundations for developing potential interventions. These approaches have brought hopes for keratoconus therapy with more safe and accessible alternative options, reducing the surgical complication and burden of limited donor corneas globally. Generally, DALK has become an alternative to PK, while minimally invasive surgery will become a major trend in the future treatment of keratoconus. And keratocyte regeneration therapies will also usher in a new era, especially for the ADSCs-based treatment, though the potential of several novel therapies for achieving effective stromal regeneration need further explorations. Certainly, further studies should be conducted to confirm the optimal therapeutic methods and conditions for keratoconus intervention, and novel approaches would be developed to control and arrest the progression of keratoconus in its much earlier stage, which might hopefully postpone or prevent an invasive corneal surgery. For keratoconus treatment, the light is shining brighter on its way.

## Data Availability

All datasets used in this study are available from the corresponding author on reasonable request.

## Abbreviations

ADSCs: Adipose tissue-derived stem cells
ESCs: Embryonic stem cells
iPSCs: Induced pluripotent stem cells
PK: Penetrating keratoplasty
DALK: Deep anterior lamellar keratoplasty
ALK: Anterior lamellar keratoplasty
EP: Epikeratophakia
BL: Bowman layer
FL-MILK: Femtosecond laser-assisted minimally invasive lamellar keratoplasty
BPCDX: Bioengineered porcine construct, double cross-linked
RHCIII: Recombinant human collagen type III
